# Corrosion Resistance of Waterborne Epoxy Resin Coating Cross-Linked by Modified Tetrabutyl Titanate

**DOI:** 10.1155/2020/1392385

**Published:** 2020-10-03

**Authors:** Lingli Xu, Zheng Chen, Fei Huang, Yinze Zuo, Xingling Shi, Xiaowei Zhou

**Affiliations:** ^1^School of Materials Science and Engineering, Jiangsu University of Science and Technology, Zhenjiang, China 212003; ^2^Jiuyang Fishing Tackle Co., Ltd., Yangzhou, China 225008; ^3^Jiangsu Gemei High-Tech Development Co., Ltd., Nantong, China 226009

## Abstract

The development of waterborne coating is essentially important for environmental protection, and cross-linking agent is of great significance for ensuring corrosion resistance of the coating. In this work, tetrabutyl titanate was modified by ethylene glycol and tris(2-hydroxyethyl) amine and used for the solidification of waterborne acrylic-epoxy resin. Fourier-transform infrared spectroscopy (FTIR) analysis revealed that the agent reacted with OH groups first to cross-link the resin preliminarily, and then, when the amount of agent was further increased, the amino groups opened epoxide rings resulting in a secondary cross-link. Field emission scanning electron microscope (FESEM) observation and electrochemical impedance spectroscopy (EIS) test found that, when the cross-linking agent was used at 6%, the coating remains intact and kept an impedance of as high as 10^8^*Ω*cm^2^ even after being immersed in NaCl solution for 30 days. Copper-accelerated acetic acid-salt spray (CASS) test confirmed that the coating containing 6% cross-linking agent provided the best protection for the carbon steel substrate.

## 1. Introduction

Due to excellent mechanical properties, mature processing technology, and low price, carbon steel becomes the most widely used engineering material in many fields. Nevertheless, corrosion has long been the problem that has not been well solved, which limits applications of carbon steels, shortens their serving life, and causes accidents and material loss [1–5]. Coating can isolate steel from the aggressive environment and has become an effective method to suppress the corrosion. Nowadays, as the call to protect the environment is growing, the research and application of waterborne coatings have been greatly developed. Among them, the epoxy resin has been widely studied and used due to its high resistance to organic solvents, strong adhesion, high electric insulation, and good thermal shock resistance [6]. However, the waterborne coating still suffers from some weakness. For example, although waterborne epoxy resin is one of the best developed waterborne coatings, its corrosion resistance is still greatly inferior to that of organic solvent coatings [7, 8]. Studies have shown that a well-designed cross-linking agent could improve the cross-linking density of waterborne resins, enhance their bonding to the matrix, and thus effectively improve the corrosion resistance of the coating. Therefore, the developing of novel cross-linking agent has become one of the research hotspots in the field of waterborne coatings.

In the previous researches on designing of agents, most scholars focused on ring-opening cross-link. For example, Lu et al. prepared a silane-modified waterborne epoxy cross-linking agent and studied the effects of the amount of cross-linking agent on performances of the cured film. They found that hardness, water resistance, and adhesion of the cured film were improved significantly [9]. Sun et al. synthesized a self-emulsified waterborne epoxy cross-linking agent of nonionic type using triethylene tetramine (TETA), and they found that epoxy resin film cured by the agent under ambient conditions showed good properties even at high staying temperature [10]. Although films cross-linked through ring-open mode usually showed good water resistance and corrosion resistance within a short period, the durability of the performance needs to be improved urgently.

The molecular structure of the cross-linking agent determines the cross-link mode of the waterborne epoxy resin. Once a proper agent was used, OH groups could also be active groups that could contribute to increasing the cross-link density of the epoxy resin. Given this, our group have developed a novel titanium ion a cross-link agent by modifying tetra-n-butyl titanate with ethylene glycol and tris(2-hydroxyethyl) amine and have evaluated its effects on the cure of epoxy ester. It was found that the agent replaced the hydroxyl groups in the epoxy ester and connected cross-link nodes in the coating and, as a consequence, improved the water resistance and corrosion resistance of the coating [11]. Due to excellent solubility in water and excellent long-term storage stability, the acrylic-epoxy emulsion has become the most sophisticated and most widely used waterborne epoxy resin [12, 13]. Therefore, this paper studied the effect of the new cross-linking agent on the cross-link behavior and corrosion resistance of the waterborne acrylic-epoxy coating.

## 2. Materials and Methods

### 2.1. Synthesis of the Agent

The titanium ion cross-linking agent was prepared in the same way stated in our previous work [11]. A distillation unit was used for synthesis and pure nitrogen was used for protection. The mole ratio of tetra-n-butyl titanate : ethylene glycol : tris(2-hydroxyethyl) amine was 1 : 1 : 2.

### 2.2. Cross-Link of Waterborne Epoxy Resin

The cross-linking agent synthesized above was added to the waterborne epoxy resin at a mass ratio of 3%, 6%, and 9%, respectively, and then the mixtures were stirred at a constant speed for 10 min. After adding water of three times the weight of the above mixture, the stirring was conducted again until the resin was evenly dispersed in the water. Cold rolled A3 mild steel sheets were used as substrate. They were polished by sandpaper of different grades until 1200#, ultrasonically cleaned with acetone and ethanol, and dried in air. The mixtures were coated onto steel sheets, respectively, by the brushing method, and then the coatings were solidified at 80°C for 3 h. Thickness of coatings was monitored by Elcometer Kairda KD 5 and was about 65 *μ*m. Coatings were coded according to the content of a cross-link agent and were ET3, ET6, and ET9 in sequence. Waterborne epoxy resin without a cross-link agent was used as control.

### 2.3. Characterizations

The FTIR spectra of the coatings were recorded with an ATR-Nicolet iS10 Spectrometer (Thermo Fisher Scientific, USA). The surface morphology of the cured film was characterized by ZEISS Merlin Compact (ZEISS, Germany). Electrochemical impedance spectroscopy (EIS) study was carried out using an electrochemical workstation (PGSTAT302N, Metrohm Autolab, The Netherlands). A standard three-electrode system was used, including sample as a working electrode, a platinum counter electrode (CE), and a saturated calomel electrode (SCE) as reference. The measurement was conducted in 3.5% NaCl solution, and an AC voltage with 10 mV amplitude (vs. SCE) was used as the imposed signal. Immersion test was carried out in 3.5% NaCl solution up to 30 days, and the results were recorded by digital camera. Copper salt-accelerated acetate spray (CASS) test was conducted according to ISO 3770-1976.

## 3. Results and Discussions

### 3.1. FTIR Analysis

**Figure 1:**
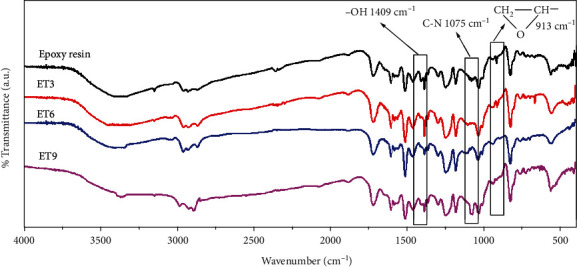
FTIR spectra of epoxy resin, ET3, ET6, and ET9 coatings.

**Figure 2:**
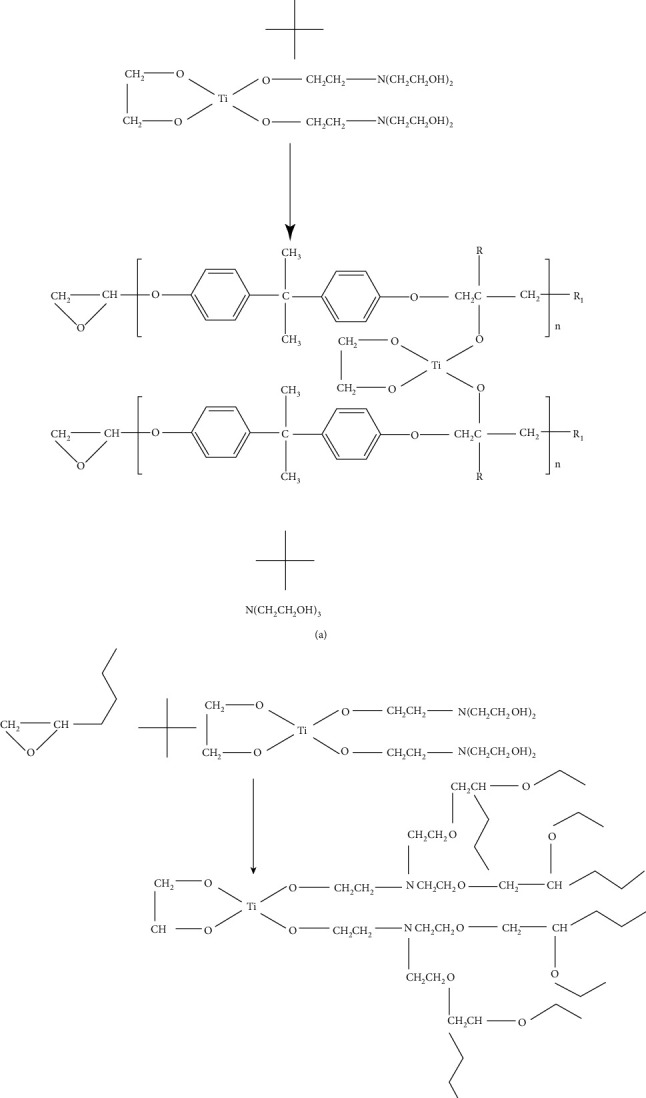
Stages involved in the reaction between epoxy ester and titanium ion curing agent. (a) Stage 1 reaction between the -OHs and titanium ion curing agent. (b) Stage 2 Reaction between the epoxide ring of and amino group.

Figure 1 shows the FTIR spectra of epoxy resin, ET3, ET6, and ET9 coatings. The absorption peak located at 913 cm^−1^ could be assigned to the stretching vibration of the saturated epoxy ring; the one at 1409 cm^−1^ represents O-H bending vibrations in epoxy resin [6, 10]. In the spectrum of the coating without the agent, both peaks could be observed clearly, which meant the cross-linking degree of resin was at a low level. When 3% of agent was used, the signal from O-H group disappeared and that from epoxy ring decreased; as amount of agent increased to 6%, both absorption peaks disappeared; when 9% of agent was used, both peaks disappeared while a strong C-N stretching vibration peak appeared. Those changes indicated that when more agent was added into resin, the cross-linking degree increased as a consequence, and agent of 6% was enough for a sufficient cross-link, whereas, when 9% of agent was used, an excessive cross-linking agent would remain in the coating. The FTIR results also suggested a chemical reaction as follows: first, the agent reacted with the O-H to cross-link resin primarily, and then, with the further increase of the cross-linking agent, the epoxy ring in the epoxy resin was opened by the -NH_3_ group, and the resin could be cross-linked further. Based on FTIR results, the reaction scheme is illustrated in Figure 2.

The double cross-linking initiated by the new agent increased cross-linking density and would definitely enhance the shielding properties of the coating, improving the corrosion resistance of the coating [11, 14, 15]. Nevertheless, if excessive cross-linking agent remains in the cured resin, it would impair the shielding property since the agent itself was hydrophilic and tends to adsorb water. Actually, the contact angle of distilled water on epoxy resin, ET3, ET6, and E9 was 61.6, 75.8, 88.0, and 80.5°, respectively.

### 3.2. Morphologies of Coatings

**Figure 3:**
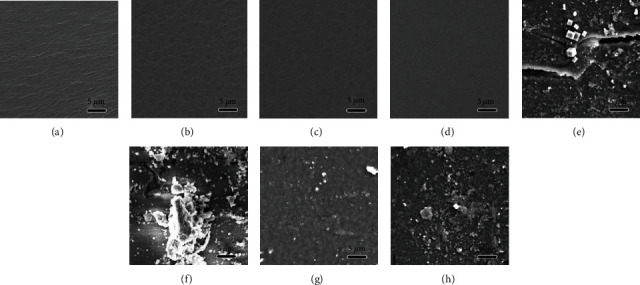
FESEM images of coating from epoxy, ET3, ET6, and ET9 before (a–d) after (e–h) 30 days of immersion in 3.5% NaCl solution.

Figure 3 shows the surface topography of coatings containing different amounts of cross-linking agent before and after immersion. It could be seen that numerous wrinkles formed on the surface of epoxy coating without agent, whereas, as the amount of agent increased, the surface of coating become smoother and smoother. When the cross-linking agent was absent, the cross-link of resin heavily depended on the evaporation of water. Since the evaporation rate of water in the surface and interior of the coating was inconsistent, the cross-link progressed asynchronously and thus caused the formation of wrinkles on the surface [16]. With the addition of cross-linking agent, evaporation of water becomes a negligible factor that promotes cross-link, and the cross-link could happen although the coating synchronously, and therefore, the wrinkles and cloud-like pattern disappeared gradually.

After 30 days of immersion, the surface morphology of the coating varied depending on the content of the cross-linking agent. Wide and deep cracks could be seen on the surface of the coating without agent while shallow erosion gullies were found on coating with 3% of agent. When 9% of agent was used, the coating surface became much rougher and showed a tendency of fragmentization. In contrast, the coating containing 6% of agent remained intact although the surface became relatively rough. It could be seen that the addition of the cross-linking agent greatly improved the water resistance of the coating; however, if the initial amount was excessive, the residual agent would cause decrease in durability in corrosive medium.

### 3.3. EIS Study

**Figure 4:**
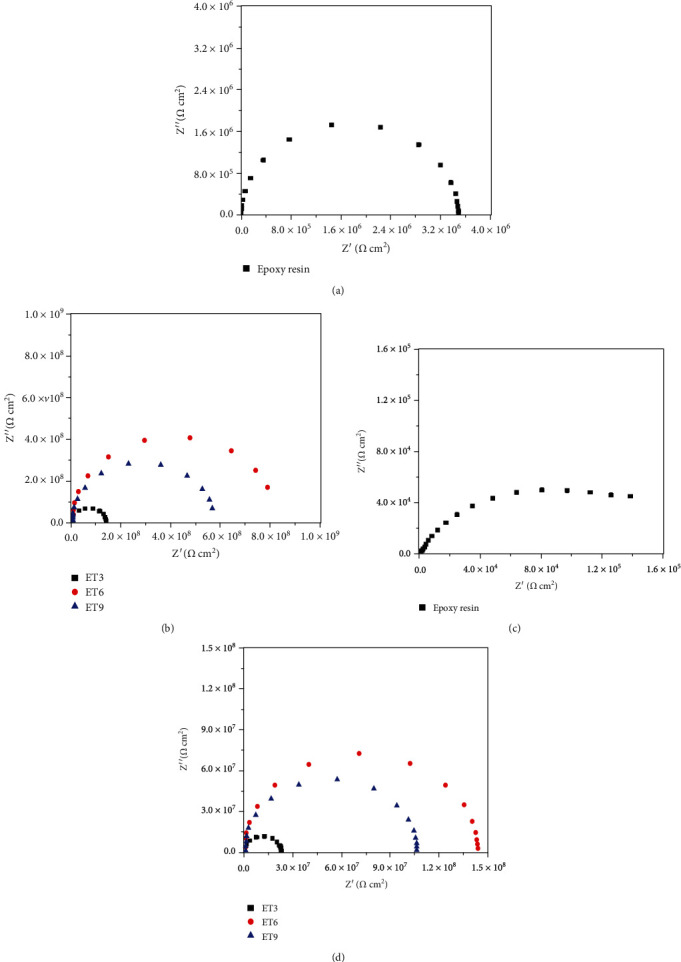
Nyquist plots of coatings after 10 days of immersion (a, b) and 30 days of immersion (c, d).

**Figure 5:**
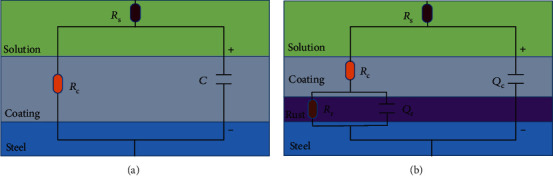
Equivalent circuits used to interpret impedance data.

EIS data obtained from different coatings are shown in Figure 4. After 10 days of immersion, all the Nyquist plots presented as semicircles, and it meant at this stage, all the coatings remained intact and behaved as a perfect barrier layer to the aggressive medium. The plot could be well fitted by the circuit model (a) in Figure 5, in which, *R*_s_ was the solution resistance, *C* was assigned to the capacitance of the coating, and *R*_c_ represented resistance of the coating. The fitting results are presented in Table 1. After 30 days of immersion, two time constants could be noticed in the Nyquist plot of coating without a cross-link agent, indicating that corrosive medium has reached the interface of coating and steel and rusting had started. At this point, the behavior of epoxy coating without a cross-link agent could be interpreted by the model (b) in Figure 5, where *Q*_C_ was assigned to CPE of polymer coating and *R*_r_ and *Q*_r_ represented resistance and CPE of the rust layer. In contrast, the plots of coating with a cross-link agent kept the shape of semicircle, and therefore, the EIS data were interpreted using the model (a) again. The fitting results are shown in Table 1 as well. According to previous reports, an epoxy coating with a resistance of 10^6^ − 10^9^ *Ω* cm^2^ was considered to have a good protection for the substrate [17–19]. It was obvious that the use of cross-linking agent increased impedances of coatings significantly, and more importantly, it could help maintaining the high impedance after 30 days of immersion.

**Figure 6:**
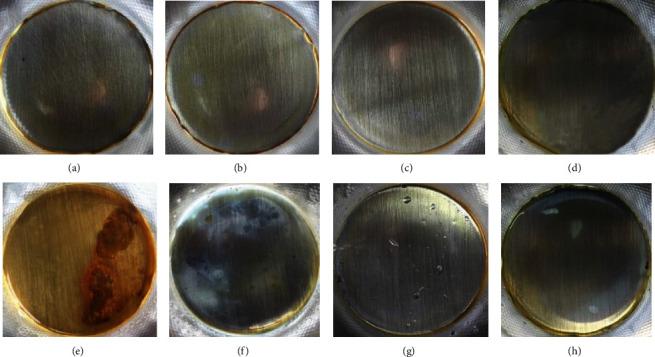
Appearance of epoxy resin, ET3, ET6, and ET9 coating before (a–d) and after (e–h) 30 days of immersion in 3.5% NaCl solution.

The appearances of the specimens coated with epoxy with different amounts of cross-link agent before and after immersed in 3.5% NaCl solution are shown in Figure 6. All the coatings were intact and almost transparent before immersion, and the freshly polished steel substrate could be seen clearly. After 30 days of immersion, rust was observed all over the steel under coating without a cross-link agent, and serious leakages could also be observed in some regions. Both ET3 and ET9 coatings remained intact, and rust was not observed; however, white cloudy areas could be seen, and this indicated that medium had penetrated into the coating deeply and might reach the substrate shortly. In contrast, ET6 coating not only protected the substrate well but also showed excellent stability in the medium that changes in the appearance could hardly be seen. The use of even very small amounts of the agent in the epoxy coating could significantly increase the cross-link density and thus helped blocking invasion of the corrosive medium [20]. However, when the content of the agent exceeds the optimum content, it would increase the water absorbability of the coating and reduce the stability and durability of the coating.

### 3.4. CAAS Test

**Figure 7:**
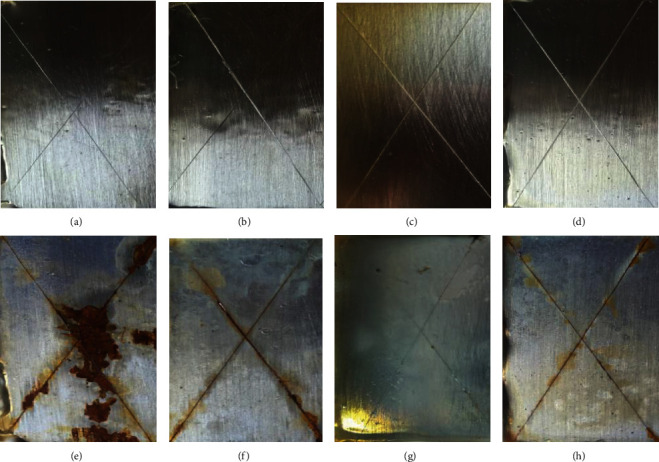
Appearances of epoxy, ET3, ET6, and ET9 coatings before (a–d) and after (e–h) 5 days of the CAAS test.

The appearances of specimens before and after 5 days of CAAS test are shown in Figure 7. For the specimen coated with epoxy without a cross-link agent, large bubbling areas could be observed, and when coating was peeled off from the steel, severe corrosion along the scratches happened and plenty rust could be seen. For ET3 coating, the corrosion products appeared along the scratches and were accompanied by blisters and slight peeling of the coating. For ET9 coating, only small blisters could be seen along the scratches, while the rest of the coating remained unaffected. It was worth noticing that the ET6 coating kept intact, nor blisters or any corrosion product could be seen along the scratches. From the above observation, the corrosion resistance of coating could be ranked in the following order incontestably: ET6 > ET9 > ET3 > epoxy. Besides, according to a system established by Abhijit et al. [21], the corrosion resistance of the coatings could be also rated clearly as shown in Table 2.

## 4. Conclusion

A novel cross-link agent was synthesized by modifying etra-n-butyl titanate with ethylene glycol and tris(2-hydroxyethyl) amine. The agent initiated double cross-link, greatly increased cross-linking density of waterborne acrylic-epoxy resin, and increased the impedance from 10^6^*Ω*cm^2^ to 10^8^*Ω*cm^2^. After 30 days of immersion, NaCl solution passed through the coating without the cross-linking agent, causing the substrate to corrode, while all the coatings with a cross-linking agent could protect the substrate well. The copper-accelerated acetic acid-salt spray test also confirmed that the adding of cross-linking agent had greatly improved protecting function of the coating. In summary, the environmentally friendly and efficient novel cross-linking agent will be promising for the application for waterborne acrylic-epoxy resin coating.

## Figures and Tables

**Table 1:** Parameters gained from fitting of the EIS data after 10 and 30 days of immersion in 3.5% NaCl solution.

Coating	*R*_c_ (*Ω* cm^2^)	*C* (F)	*Q*_c_ (*Ω*^−1^s*^n^*cm^−2^)	*n*	*R*_r_ (*Ω* cm^2^)	*Q*_r_ (*Ω*^−1^s*^n^*cm^−2^)	*n*	Equivalent circuit model	Immersion duration/days
Epoxy	3.5 × 10^6^	2.2 × 10^−8^	—	—	—	—	—	A	10
ET3	1.0 × 10^8^	1.4 × 10^−9^	—	—	—	—	—	A	
ET6	8.3 × 10^8^	2.6 × 10^−10^	—	—	—	—	—	A	
ET9	5.7 × 10^8^	2.1 × 10^−9^	—	—	—	—	—	A	

Epoxy	1.3 × 10^5^	—	1.8 × 10^−9^	0.61	575	1.1 × 10^−5^	0.52	B	30
ET3	2.3 × 10^7^	2.5 × 10^−9^	—	—	—	—	—	A	
ET6	1.5 × 10^8^	1.1 × 10^−9^	—	—	—	—	—	A	
ET9	1.1 × 10^8^	1.3 × 10^−9^	—	—	—	—	—	A	

**Table 2:** Corrosion resistance rating of coatings after 5 days of CAAS.

Coatings	Rating
Epoxy resin	1
ET3	4
ET6	6
ET9	5

## Data Availability

The FTIR, SEM, EIS, and photos used to support the findings of this study are included within the article.
